# Personality Traits in College Students and Caregiving for a Relative with a Chronic Health Condition

**DOI:** 10.1155/2016/3650927

**Published:** 2016-09-06

**Authors:** Michael A. Trujillo, Paul B. Perrin, Aaliah Elnasseh, Bradford S. Pierce, Melody Mickens

**Affiliations:** Virginia Commonwealth University, Richmond, VA, USA

## Abstract

The purpose of this study was to investigate among college students the relationship between personality traits and willingness to care for a relative with a chronic health condition. 329 undergraduate students completed an online questionnaire. Hierarchical multiple regressions found that after controlling for demographics personality traits explained 10% of the variance in willingness to provide emotional care, 7% in instrumental care, and 7% in nursing care. Within these models, greater empathy was uniquely associated with willingness to provide emotional, instrumental, and nursing care for a family member in the future. Similarly, participants with high agreeableness were more willing to provide emotional care, and participant older age was a unique predictor of instrumental care. The results can help shape research on interventions that incorporate perspective taking, motivational interviewing, and training in life skills as a means of boosting college students' willingness to provide care for a relative with a chronic health condition.

## 1. Introduction

By 2050, the number of people over the age of 65 is projected to increase from an estimated 524 million to nearly 1.5 billion, representing 16% of the world's population [1]. Many of these individuals and their families will experience challenges as older adults learn how to manage and cope with chronic health problems such as cancer, heart disease, and diabetes that require long-term care [1]. Over the past decade, significant increases in healthcare costs have led to greater utilization of and increased need for informal caregiving from family members who provide emotional support, assistance with activities of daily living (e.g., bathing, grooming, and feeding), and instrumental support (e.g., paying bills, shopping, and assistance with medication) for individuals with chronic illness and disabilities [2, 3].

Trends of older adults living longer with chronic illnesses have coincided with younger adults (i.e., 18–25-year-olds) assuming caregiving responsibilities for family members [2], especially since marriage and childbearing have been delayed and college enrollment overall in the young adult population has increased [4]. Young adults comprise 12–18% of adult caregivers, and the average age of a young adult caregiver is 21 years [4], suggesting that many young caregivers are college-age. Although young adult caregivers cannot be automatically equated with college students, as social selection processes may predispose certain young adults to take on a family caregiving role, the National Center for Education Statistics estimates that 40 percent of the US population between the ages of 18 and 24 is enrolled in degree-granting postsecondary institutions, representing a sizeable group of young adults who are or could become caregivers [5]. One study of young adult caregivers, for example, found 38 percent of participants caring for grandparents reported completing some college [6]. Although little is known about undergraduate students acting as family caregivers, research has found that caregiving by college students has been associated with increased burden in transitioning to college, reduced social engagement, and greater stress and depression [6, 7]. Similarly, college caregivers may experience additional stress because they may be relatively inexperienced with providing care [8], which may pose more challenges for them in fulfilling their duties.

The more general literature outside of college students has shown that factors such as caregiving history, SES, and the availability of support programs, along with mediators such as social support systems and personal coping responses, may influence an individual's ability to handle the stressors that accompany transitioning to the caregiving role [9]. There is considerable variety between families and individual members in their ability to recognize and plan for the caregiving needs of others. Individuals who anticipate and plan for the needs of older adult family members are more satisfied with the amount of family discussion and planning than those who ignore those needs [10]. Beliefs and personality characteristics play a meaningful role in an individual's anticipation of and decision to adopt a caregiving role. For example, family members with an internal locus of control tend to anticipate and prepare for the caregiving needs of their parents and grandparents [11]. Those with an avoidant attachment style perceive caregiving as a burden and are less willing to provide future care for parents [12]. In contrast, adult children who possess more filial obligation and display attachment to parents are more likely to commit to future help [13].

Despite the research examining predictors of adjustment to becoming a caregiver and the more specific research beginning to identify the widespread nature of caregiving duties among college students, no research has examined the personality characteristics that might predispose college students to assuming a caregiving role. This is unfortunate because the more general research has identified personality variables as playing a major role in shaping caregiving experiences [14, 15]. A well-established measure of personality is the Five-Factor model, which includes the dimensions extraversion, agreeableness, conscientiousness, neuroticism, and openness to experience [16]. The Big Five personality traits have been found to be related to caregiver evaluation of care tasks, use of coping strategies, and evaluation of the care recipient's impairment [17, 18].

Extraversion in particular has been associated with positive experiences in providing care and a greater ability to recognize the benefits of caregiving [19, 20]. Also, caregiver agreeableness and conscientiousness have been related to a better relationship with the care recipient [21], as well as to increased benefit finding [19]. Caregiver neuroticism, on the other hand, has been associated with more mental health problems [18], increased susceptibility to care-related stressors [19, 22], and decreased salutogenic behaviors [18]. There is also evidence suggesting extraversion and neuroticism may predict caregivers' level of optimism concerning their future health [23]. Finally, caregiver openness has been related to awareness for future care needs and more gathering of care-related information [24].

Outside of the Big Five model, caregivers with greater empathy report higher life satisfaction and less depression, as well as appraising caregiving as less threatening and stressful [25]. Broadly defined, empathy is the ability to correctly perceive another's feelings [26]. A proposed model of caregiver compassion [27] suggests personality characteristics such as empathy, level of intimacy, and attachment style moderate the level of compassion people feel in response to another's suffering, resulting in (or inhibiting) various helping behaviors [12]. As individuals develop empathy, they learn to recognize and feel what others feel before developing the capacity to maintain emotional distance from another's internal experience while retaining the ability to recognize another's feelings [25]. Caregivers in the early stages of empathy development may quickly feel overwhelmed by the emotional experience they share with the recipient [25]. In contrast, greater empathy has been associated with better provision of support by caregivers and reduced anxiety [28].

Given the aging demographic trends in the US and the increasing likelihood that today's college-age young adults will one day provide care for their relatives with a chronic health condition [2], it is extremely important to examine the factors that might predict their willingness to provide care. While previous studies have examined the relationship between a number of personality traits and different aspects of caregiving [15], including how personality traits are associated with assuming and maintaining a caregiving role [29], limited research to date has investigated the relationship between personality traits and one's willingness to provide care for a family member with a chronic health condition, especially in a young adult population that will be increasingly likely to do so.

*Objective*. The purpose of the current study is to examine whether the Big Five personality traits and empathy are associated with college students' willingness to provide care for a family member with a chronic condition. First, it is hypothesized that empathy, agreeableness, and conscientiousness will be positively associated with willingness to provide emotional care. Research has suggested that agreeableness and conscientiousness are both related to a better relationship with the care recipient [21], as well as to increased benefit finding [19]. Second, it is hypothesized that openness will be positively associated with willingness to provide instrumental care. Previous research has shown that openness is related to awareness of future care needs and more gathering of care-related information [24]. Third, it is hypothesized that neuroticism will be negatively associated with willingness to care more generally. Prior research indicates caregiver neuroticism is associated with more mental health problems [18], increased susceptibility to care-related stressors [19, 22], and decreased salutogenic behaviors [18]. And finally, it is hypothesized that extraversion and empathy will be positively associated with general willingness to care. Research has shown that extraversion is related to positive experiences in providing care and to recognizing the benefits of caregiving [19, 20].

## 2. Method

### 2.1. Participants

Data collection occurred from March through May of 2014. Participants were undergraduate students enrolled in psychology courses at a large urban university. All participants were over age 18 and enrolled in a psychology course at the university. Initially, 343 students completed the study. Only participants who answered at least five of seven (71%) reliability-check items correctly were included in the current study with 13 respondents omitted for this reason (see Table 1 for a description of participant demographics). Additionally, one participant was excluded who was identified as intersex in order to be able to include gender as a covariate in our analyses, yielding a final sample size of 329.

### 2.2. Measures

#### 2.2.1. Social Class

Participants indicated social class by answering, “What is your family's social class? Upper Class: $200,000 & up (CEOs, Politicians); Upper Middle Class: $60,000–199,999 (Professionals); Lower Middle Class: $30,000–59,999 (Professional Support & Sales); Working Class: $15,000–29,999 (Clerical, Service, & Blue Collar); Lower Class: $7,000–14,999 (Part-time & Unemployed).” Responses were coded 5, 4, 3, 2, and 1 respectively. Given that income was the primary component assessing social class, it was treated as a continuous variable in all analyses.

#### 2.2.2. Employment Status

Participants indicated employment status by answering, “Are you currently employed? (Please select one). No. Yes, Full Time (at least 36–40 hours/week). Yes, Part-Time (no more than 35 hours/week).” Responses were coded as 1, 3, and 2 respectively.

#### 2.2.3. Prior Caregiving Experience

Participants answered “Yes” or “No” to the following question, “In the past (but no longer), did you provide unpaid informal care to a relative or friend with a chronic health condition lasting three months or longer?” “No” responses were coded as 0 while “Yes” responses were coded as 1.

#### 2.2.4. Gender

Participants indicated gender by answering, “What gender label best describes you (select one)? Man, Woman, Intersex, Transman, Transwoman.” Responses were coded as 1, 2, 3, 4, or 5, respectively. All but one participant were identified as either man or woman and as such gender was treated as a dichotomous variable.

#### 2.2.5. Age

Participants reported their age in response to “How old are you (in years)?”

#### 2.2.6. Willingness to Care (WTC)

The WTC scale is a 30-item measure assessing an individual's attitude toward providing instrumental, nursing, and emotional support for individuals with a chronic condition [30]. The original scale was intended to assess willingness to care for a person with HIV/AIDS; thus the directions were modified such that “person with HIV/AIDS” was replaced by “a family member that you might have to provide care for at some point because they have a chronic illness, injury, or disability.” Individual caregiving items within the questionnaire are general in nature (e.g., providing encouragement, cleaning up, and paying for medicines) and not specific to persons with HIV/AIDS, so no further modifications were necessary. In order to prevent alternative explanations that may explain nonwillingness of participants to take over care responsibilities, such as an inability or financial circumstances, we instructed participants to “assume that you are able to provide the following types of care. Select the response that best shows how willing you are to do each one.” Additionally, participants were told that a “chronic health condition is a disease or a disability that lasts for THREE MONTHS OR LONGER.” The WTC has four scores: a global willingness-to-care score as well as three mean subscale scores including emotional care (providing emotional support such as encouraging someone who feels hopeless, e.g., to “listen to someone's concerns about death or dying”), instrumental care (assisting with everyday tasks such as preparing meals for someone, e.g., to “bring home groceries for someone”), and nursing care (measuring physical caretaking activities such as helping someone take medicine, e.g., to “clean up after someone who has lost bowel or bladder control”). Higher scores indicate greater willingness to provide care. The WTC score has high reliability for each subscale (alphas range: .84–.91) [30].

#### 2.2.7. Big Five Inventory-10 (BFI-10)

The BFI-10 is a 10-item scale with an optional 11th item derived from the Big Five Inventory-45 measuring the “Big Five” factors of personality: neuroticism, extroversion, openness, agreeableness, and conscientiousness. The 11-item version was used for the current study, producing subscales composed of two items except for agreeableness, which was composed of three items [31]. Higher scores indicate higher levels of the personality trait. The BFI-10 has shown good convergent validity [31]. In the current study, Pearson product moment correlations were calculated for the extraversion (*r* = .391), conscientiousness (*r* = .362), and neuroticism (*r* = .348) items, which were all significant (*p* < .001), as were the correlations among the three items composing the agreeableness subscale (*r*: .213–.256; *p* < .001). The bivariate correlation for the items assessing openness was not significant (*r* = .058, *p* = .290) suggesting a potential issue with the internal consistency for this subscale.

#### 2.2.8. Toronto Empathy Questionnaire (TEQ)

The TEQ is a 16-item scale that assesses the ability to perceive the emotional state of another person and respond sympathetically. Higher scores indicate higher empathy [26]. The TEQ has good internal consistency (Cronbach's alpha = .85) and convergent validity [26].

### 2.3. Procedure

Study personnel emailed psychology course instructors with information describing the study. Instructors who were willing to assist provided students with study information and a link to the survey. Interested students reviewed and completed an online consent prior to participation and completed the survey by submitting their responses using the online platform. Participants received extra credit points at the instructor's discretion, but not totaling more than 1% of their overall grade. This study received institutional review board approval by the authors' institution prior to recruitment.

### 2.4. Data Analysis

Preliminary analyses examined correlations within each of the WTC subscales, the BFI subscales, and the TEQ. Three hierarchical multiple regressions investigated the extent to which personality traits (neuroticism, openness, extraversion, agreeableness, and conscientiousness) and empathy were associated with each of the willingness-to-care subscales (emotional care, instrumental care, and nursing care). Prior to running primary analyses, a series of bivariate correlations between the three types of willingness to care and demographic characteristics including age, gender, family socioeconomic status, and employment status were conducted to identify important covariates to include in the models, as demographics have previously been shown to be associated with care provision [32, 33]. As prior work has found that individuals with less time available due to their employment status (e.g., families with dual earners compared to single earners) provide less care [32], we treated employment status as a continuous variable to assess if this phenomenon extended to willingness to provide care. Given that much of the prior work has not been conducted with college-age samples, only demographic characteristics that were significantly associated with outcome variables in the current sample would be included in the final analyses. Age, gender, and employment status were shown to be associated with the outcomes in the bivariate correlations and were included as covariates in all analyses (Table 2). In each of the three regressions, demographics were entered in the first step, with all of the personality variables as well as empathy entered in the second step. The three willingness-to-care subscales were entered as the dependent variable in each regression. To explore if age, gender, or employment status interacted with personality traits, a series of exploratory analyses were conducted. Interactions between each personality trait and age and each demographic characteristic were included in the third step predicting each outcome variable and are summarized following the primary results for each criterion variable.

## 3. Results

### 3.1. Normality Assumptions

Normality assumptions were assessed prior to running analyses. For all five personality subscales (neuroticism, openness, extraversion, agreeableness, and conscientiousness), empathy, and nursing care, normality assumptions were met. However, emotional and instrumental care had skewness of −3.82 and −1.46 and kurtosis of 18.62 and 2.60, respectively, and thus did not meet the assumption of normality. To address these violations, a reflection of emotional and instrumental care data was performed via log transformation and produced new skewness values of .70 and −.40 and kurtosis values of −.29 and −1.07 for emotional and instrumental care, respectively. An assessment of VIF and tolerance statistics indicated no multicollinearity was present among the variables. Additionally, an examination of bivariate correlations indicated no correlations greater than .70.

### 3.2. Correlation Matrix

A series of correlations were conducted to examine the bivariate relationships between all variables (Table 3). All willingness-to-care variables were positively associated with each other, as well as being positively related to empathy and personality traits of agreeableness and conscientiousness. Willingness to care was not related to extraversion, neuroticism, or openness. Extraversion was positively related to agreeableness and empathy but negatively associated with neuroticism, while agreeableness was positively related to conscientiousness and empathy. Additionally, neuroticism and conscientiousness were negatively related, while empathy was positively related, to all personality traits except for neuroticism and openness. All other bivariate relationships were not statistically significant.

### 3.3. Emotional Care

In the first hierarchical multiple regression (Table 4), NEO personality traits and empathy were regressed onto willingness to provide emotional care after accounting for age, gender, and employment status. Demographics were entered into the first step, for which the overall model was significant, *F*(3,325) = 3.21, *p* < .05, and *R* ^2^ = .03. No demographic characteristics produced any unique effects (all* p* > .053). All personality variables as well as empathy were entered in the second step which was also significant, *F*(9,319) = 5.41, *p* < .001, and *R* ^2^ = .13, with a significant increase in the amount of variance explained in emotional care, Δ*F*(6,320) = 6.35, *p* < .001, and Δ*R* ^2^ = .10. The personality trait of agreeableness as well as empathy was uniquely and positively associated with emotional care. All other personality variables were not independently related to emotional care (all* p* > .184). This suggests that greater agreeableness and empathy are associated with greater willingness to provide emotional care.

To explore if age, gender, or employment status interacted with each personality trait, interaction terms with each of these demographic variables and the personality traits were included in step three. The overall model was significant, *F*(27,301) = 2.13, *p* < .01, and *R* ^2^ = .16; however, there was no significant increase in explainable variance Δ*F*(18,301) = 0.55, *p* = .931, and Δ*R* ^2^ = .03. There were also no significant interactions between personality traits by age (all* p* > .257), gender (all* p* > .240), or employment status (all* p* > .186).

### 3.4. Instrumental Care

In the second hierarchical multiple regression (Table 5), demographics were entered in the first step, yielding a significant test, *F*(3,325) = 6.21, *p* < .001, and *R* ^2^ = .05. Both age and employment status were uniquely and positively associated with instrumental care. In the second step, empathy and all personality variables were entered, producing an overall significant model, *F*(9,319) = 4.81, *p* < .001, and *R* ^2^ = .12. There was also a significant increase in the amount of variance explained in instrumental care, Δ*F*(6,319) = 3.94, *p* < .001, and Δ*R* ^2^ = .07. After accounting for demographics, empathy was independently positively associated with instrumental care with age maintaining significance. No NEO personality traits were associated with instrumental care (all* p* > .187) and employment status dropped in significance in follow-up analyses (*p* = .090). This finding suggests that greater empathy and older age are related to greater willingness to provide instrumental care.

**Figure 1:**
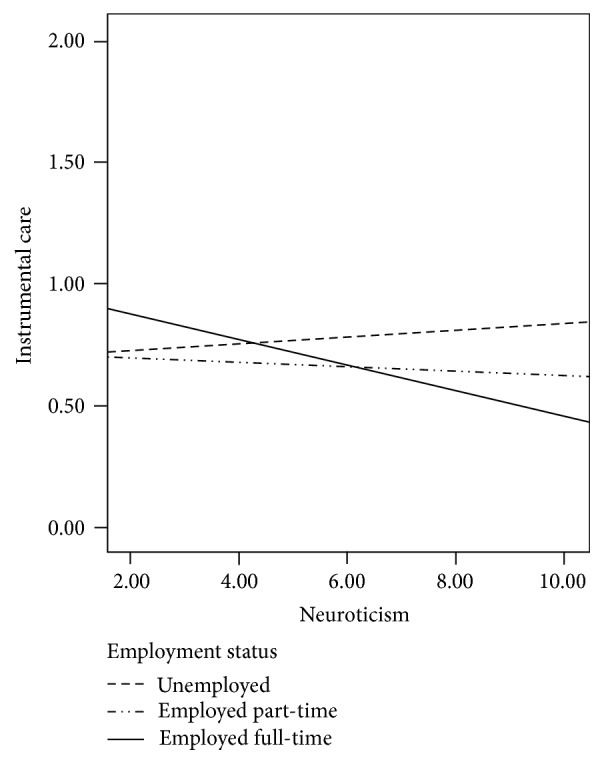
Interaction of employment status by neuroticism on willingness to provide instrumental care.

As part of the exploratory multiple regressions, separate interaction terms between age, gender, and employment status with personality characteristics were included in step three. The overall model was significant, *F*(27,301) = 1.990, *p* < .01, and *R* ^2^ = .15; however, there was no significant increase in explainable variance Δ*F*(18,301) = 0.631, *p* = .874, and Δ*R* ^2^ = .03. There was a significant interaction of employment status by neuroticism (*β* = .44, *p* = .030), such that individuals who were more neurotic and were employed full-time were less likely to provide care than unemployed or part-time employed individuals (Figure 1). All other interactions with personality traits (all* p* > .380) as well as interactions between personality traits by age (all* p* > .267) and by gender (all* p* > .089) were not significant.

### 3.5. Nursing Care

In the third regression (Table 6), demographics were entered in the first step, which was not significant, *F*(3,325) = 2.45, *p* = .063, and *R* ^2^ = .02. All personality traits and empathy were entered into the second step, which was significant, *F*(9,319) = 3.61, *p* < .001, and *R* ^2^ = .09, with a significant increase in the amount of variance explained, Δ*F*(6,319) = 4.12, *p* < .01, and Δ*R* ^2^ = .07. Only empathy was uniquely related to willingness to provide nursing care. No NEO personality traits or demographic characteristics were uniquely related to willingness to provide nursing care (all* p* > .08). This suggests that greater empathy was associated with greater willingness to provide nursing care.

Similar to the third step in the first two exploratory multiple regressions, separate interaction terms between age, gender, and employment status with personality traits were included in step three. The overall model was significant, *F*(27,301) = 1.74, *p* < .05, and *R* ^2^ = .14; however, there was no significant increase in explainable variance Δ*F*(18,301) = 0.82, *p* = .672, and Δ*R* ^2^ = .04. All interactions of personality traits by age (all* p* > .366), by gender (all* p* > .100), and by employment status (all* p* > .182) were not significant.

## 4. Discussion

With more adults living to an older age and with chronic health conditions, younger adults are assuming greater roles as family caregivers. Though many young caregivers are in college, no studies have examined the association between personality traits and willingness to provide care in a college-age population. As such, the purpose of this study was to investigate the relationship between the Big Five personality traits and empathy and willingness to provide care for a relative with a chronic health condition in a college-aged sample. It was hypothesized that empathy, agreeableness, and conscientiousness would be positively associated with willingness to provide emotional care, that openness would be positively associated with willingness to provide instrumental care, that neuroticism would be negatively associated with willingness to care more generally, and that extraversion and empathy would be positively associated with general willingness to care. A series of hierarchal multiple regressions found that after controlling for demographics, personality traits explained 10% of the variance in willingness to provide emotional care, 7% in instrumental care, and 7% in nursing care. Within these models, greater empathy was uniquely associated with willingness to provide emotional, instrumental, and nursing care for a family member in the future. Similarly, participants with high agreeableness were more willing to provide emotional care, and older age was a unique predictor of instrumental care.

The finding that college students who exhibit a greater level of empathy are more willing to care for a family member with a chronic health condition is in line with previous research indicating empathy is a source of altruistic motivation. According to the empathy-altruism hypothesis, the motivation to help is altruistic to the degree that it is evoked by an empathic emotional response, such that empathic concern can lead to altruistic motivations to help others [34, 35]. By extension, dispositional empathy has been shown to be predictive of greater empathic concern [36]. This suggests that those who exhibit more empathic characteristics may have a greater tendency for empathic responding and subsequently are more motivated to help or care for a family member, as found in the current study. Given that empathy was uniquely predictive of all three willingness-to-care subscales, participants who were more empathic may have been more willing to help or provide care irrespective of the type of care, which is supported by numerous studies linking empathic concern to various helping behaviors [37, 38].

Agreeableness was also identified as being uniquely and positively associated with willingness to provide emotional care for a family member with a chronic health condition. Broadly, agreeableness has been argued to be representative of more humanitarian aspects of personality including nurturance and altruism [39] and has long been identified as personality disposition positively related to volunteering [40] and prosocial behaviors [41]. This suggests that people who are more agreeable may be better able to provide emotional support such as being encouraging, caring, and comforting, as well as more inclined to engage in helping behavior. This is in line with prior research outlining that nurses with greater levels of agreeableness are likely to utilize positive emotional social support [42]. Additionally, previous work indicates that those who exhibit greater levels of agreeableness are more likely to perceive the caregiving role as meaningful and positive [19]. Within the context of the previous literature, in the current study, those scoring higher on agreeableness may have been better able to find the caregiving role as important and beneficial and therefore were more willing to provide care.

Female gender, older age, and a higher level of employment were associated with more willingness to provide various types of care in the bivariate correlation matrix. Although these findings are generally in line with the previous literature [32], it is important to note that all of these effects were small-sized in the matrix, with the statistically significant correlations indexing 1.2% (e.g., age and emotional care) to a maximum of 2.9% (e.g., age and instrumental care) of shared variance. And when entered into the multiple regressions along with the personality predictors in each model's step two, none of these demographics remained significant predictors except for age predicting willingness to provide instrumental care (*β* = .14), which just surpassed the *α* = .05 threshold. As a result, these demographic effects should largely be seen as negligible and only statistically significant in the bivariate correlations because of the current study's large sample size. However, a significant interaction of employment status by neuroticism emerged, whereby college students who were more neurotic and were employed full-time were less likely to be willing to provide instrumental care than unemployed or part-time employed individuals. A potential interpretation of this effect is that college students who are working full-time, in combination with being high in neuroticism, may feel particularly overwhelmed by their employment demands and as a result may not have the personal resources necessary to be devoted to providing instrumental care for a family member with a chronic condition. However, this effect should be interpreted with caution given that 54 demographics by personality interactions were examined in an exploratory fashion as predictors (three demographics by six personality traits, for each of the three willingness-to-care criterion variables), and this was the only interaction to reach statistical significance. This exploratory approach was highly prone to familywise error, so this effect should be examined in future studies before definitive interpretations are made.

Although results did not support our hypotheses of positive relationships between willingness to care with extraversion and openness, it is possible that the substantial amount of variance accounted for by empathy eclipsed the effect these personality aspects had on willingness to care. Additionally, it is worth noting that the correlation of the two items composing the openness subscale was relatively low and not significant, calling into question the internal consistency of this subscale in the current sample. While this scale has been previously validated among a college-aged sample and the subscale intercorrelations from the current sample are in line with those from the validated study [31], it is possible that there are additional characteristics that may have impacted the potential reliability of the openness subscale in particular and prevented it from showing an effect on willingness to care.

Though individuals with higher levels of extraversion have been shown to recognize the benefits of caregiving and tend to report positive experiences in providing care [19, 20], these rewards may not be apparent or compelling enough for extraverted college students. Research indicates potential caregivers perceiving fewer gains than losses as a caregiver may be less likely to adopt a caregiving role [43]. For extraverted college students in particular (and perhaps in comparison to caregivers from other age groups or population segments), the losses to one's social life or losses to the progression in one's academic pursuits may be fairly dramatic, and as a result some extraverted college students may be less willing to make sacrifices in their social lives in order to provide care given the extremely social nature of college settings.

## 5. Clinical Research Implications

The results of this study suggest that research on interventions attempting to increase empathy or to make it more salient may be particularly useful in exploring how to increase college students' willingness to provide care. In an intervention aimed to improve peer counseling skills in students, Hatcher et al. [44] provided instruction in nonjudgmental and empathic listening, facilitative feedback, and self-observation through role play. College students in the intervention group showed significant increases in empathic concern and perspective taking suggesting this type of intervention that targets empathic communication may be especially helpful with college students, as they may become better able to identify as the potential family member with a chronic health condition and thus more willing to provide care.

In terms of the current study's finding that agreeableness was associated with willingness to provide emotional care, intervention research making salient for college students the values of being cooperative, kind, and considerate (all facets of agreeableness) in the context of the family system may be a helpful direction. Such research could involve interventionists designing motivational interviewing techniques [45] that attempt to help college students identify discrepancies between these aspects of their personality and their actual behaviors of providing care for their family member with a health condition. Additionally, research on training that target daily life skills such as food preparation, financial management, and home maintenance may help the field better learn how to improve college student's self-efficacy in the provision of care and perhaps resultantly willingness to do so in the future. Similarly, college students who are better equipped with the necessary skills to care for themselves may be better positioned and more willing to care given greater personal resources (e.g., energy, social support) to enact informal care.

## 6. Limitations and Future Directions

There are some limitations that should be considered when interpreting the findings of this study. First, participants were asked to anticipate their willingness to provide care for a relative but were not asked to identify the person, the likelihood of actually becoming a caregiver, or the quality of their relationship to this family member. The relationship of a participant to particular family members (e.g., niece/nephew, child, and cousin), the quality of that relationship, and the likelihood of actually taking on a caregiving role with that person could indeed influence one's self-reported willingness to care. Research should ask participants specifically whom they are thinking about providing care for and what the quality of that relationship is, as these variables could make one more or less apt to provide care for the family member. Additionally, data on the severity of the illness, injury, or disability imagined by the participant were not collected and may impact willingness to provide care. Related to this, participants were asked to imagine someone that they “might have to provide care for at some point because they have a chronic illness, injury, or disability.” The definition of “chronic illness, injury, or disability” encompasses a wide range of issues and differing care demands and “at some point” covers a wide time-frame. As a result, responses may vary depending on what a respondent imagined based on this prompt. Future research may benefit from including more specific language regarding the potential provision of care by participants in the future. Family members with more severe injuries or illnesses, for instance, dementia, require greater care on behalf of the caregiver than others do, which may impact how much and what type of care they are willing to provide. Furthermore, issues related to familial responsibility were also not assessed. Students who have a greater sense of obligation and/or lack of choice to care for those in the family network may be more or less willing to care than those who perceive fewer obligations [43]. Finally, the sample consisted entirely of psychology students and was overrepresented by participants from middle-class families and women and thus may not generalize to all college students. Future studies should assess relationship quality between the potential caregiver and future care recipient as well as familial responsibility in an effort to better understand the effects of personality on willingness to provide care for a relative with a chronic health condition.

## 7. Conclusions

This study adds to the growing body of research assessing willingness to provide care for future family members by outlining specific personality traits among college students that may be influential for future care. With a growing population of older adults, young adults are primed to become caregivers in the near future. The results of this study identifying agreeableness, empathy, and age as being important for one's potential for future caregiving can help shape future interventions especially those that incorporate perspective taking and training in life skills as a means of boosting empathic concern and subsequently greater willingness to care.

## Figures and Tables

**Table 1:** Summary of participant characteristics.

Characteristics	*N*	%
Age, M (SD)	21.6 (4.29)	
Gender		
Male	95	28.9
Female	234	71.1
Race/ethnicity		
White, Caucasian	142	43.2
Black, African American	81	24.6
Asian, Asian American	55	16.7
Hispanic or Latino	21	6.4
Mixed	30	9.1
Family social class		
Lower class	12	3.6
Working class	34	10.3
Lower middle class	84	25.5
Middle class	181	55.0
Upper class	18	5.5
Employment status		
Unemployed	160	48.6
Part-time (<35 hrs/wk)	139	42.2
Full-time (36–40 hrs/wk)	30	9.1
Prior caregiving experience		
Yes	80	24.3
No	249	75.7

**Table 2:** Correlations among willingness to provide care and demographic variables.

	Age	Gender	Employment status	Social class
Emotional care	.11^*∗*^	.12^*∗*^	.08	.02
Instrumental care	.17^*∗∗*^	.12^*∗*^	.11^*∗*^	.05
Nursing care	.13^*∗*^	.09	.05	.00

*Note.* ^*∗*^ *p* < .05 and ^*∗∗*^ *p* < .01, two-tailed. Correlations with continuous demographics were calculated as Pearson and with dichotomous demographics as point-biserial.

**Table 3:** Correlations among willingness to provide care variables, NEO, and empathy.

	1	2	3	4	5	6	7	8
(1) Emotional Care	—							
(2) Instrumental care	.66^*∗∗∗*^	—						
(3) Nursing care	.60^*∗∗∗*^	.68^*∗∗∗*^	—					
(4) Extraversion	.11	.05	.03	—				
(5) Agreeableness	.25^*∗∗∗*^	.18^*∗∗*^	.19^*∗∗*^	.14^*∗*^	—			
(6) Conscientiousness	.17^*∗∗*^	.16^*∗∗*^	.17^*∗∗*^	.11	.19^*∗∗*^	—		
(7) Neuroticism	.01	.01	.03	−.16^*∗∗*^	−.10	−.11^*∗*^	—	
(8) Openness	.01	.03	.05	.05	.06	.07	−.06	—
(9) Empathy	.31^*∗∗∗*^	.28^*∗∗∗*^	.25^*∗∗∗*^	.11^*∗*^	.44^*∗∗∗*^	.21^*∗∗∗*^	.09	.07

*Note*.  ^*∗*^ *p* < .05, ^*∗∗*^ *p* < .01, and ^*∗∗∗*^ *p* < .001, two-tailed.

**Table 4:** Hierarchical multiple regression: association between demographics, personality traits, empathy, and willingness to provide emotional care.

Variable	Model 1			Model 2		
	*B*	SE *B*	β	*B*	SE *B*	β
Age	.01	.01	.11	.01	.01	.07
Gender	.10	.05	.11	.06	.05	.07
Employment	.02	.02	.05	.01	.02	.02
Extraversion				.01	.01	.06
Agreeableness				.03	.01	.14^*∗∗*^
Conscientiousness				.02	.01	.07
Neuroticism				.00	.01	.01
Openness				.00	.01	.02
Empathy				.01	.00	.20^*∗∗∗*^
*R* ^2^			.03^*∗*^			.13^*∗∗∗*^

*Note*. *N* = 329; SE = standard error.

^*∗*^ *p* < .05, ^*∗∗*^ *p* < .01, and ^*∗∗∗*^ *p* < .001, two-tailed.

**Table 5:** Hierarchical multiple regression: association between demographics, personality traits, empathy, and willingness to provide instrumental care.

Variable	Model 1			Model 2		
	*B*	SE *B*	*β*	*B*	SE *B*	*β*
Age	.02	.01	.17^*∗∗*^	.02	.01	.14^*∗*^
Gender	.09	.06	.09	.05	.06	.04
Employment	.06	.03	.12^*∗*^	.04	.03	.09
Extraversion				.00	.01	.02
Agreeableness				.02	.01	.07
Conscientiousness				.02	.02	.07
Neuroticism				.00	.01	.01
Openness				.00	.02	.01
Empathy				.01	.00	.19^*∗∗*^
*R* ^2^			.05^*∗∗*^			.12^*∗∗∗*^

*Note*. *N* = 329; SE = standard error.

^*∗*^ *p* < .05, ^*∗∗*^ *p* < .01, and ^*∗∗∗*^ *p* < .001, two-tailed.

**Table 6:** Hierarchical multiple regression: association between demographics, personality traits, empathy, and willingness to provide nursing care.

Variable	Model 1			Model 2		
	*B*	SE *B*	*β*	*B*	SE *B*	*β*
Age	.26	.12	.12^*∗*^	.20	.12	.09
Gender	1.63	1.15	.08	.73	1.19	.04
Employment	.12	.55	.01	−.13	.54	−.01
Extraversion				−.03	.27	−.01
Agreeableness				.48	.29	.10
Conscientiousness				.56	.32	.10
Neuroticism				.18	.26	.04
Openness				.21	.30	.04
Empathy				.20	.08	.16^*∗*^
*R* ^2^			.02			.09^*∗∗∗*^

*Note*. *N* = 329; SE = standard error.

^*∗*^ *p* < .05, and ^*∗∗∗*^ *p* < .001, two-tailed.
